# Heat-induced necrosis after bronchial thermoplasty: a new concern?

**DOI:** 10.1186/s13223-018-0252-y

**Published:** 2018-06-25

**Authors:** Francesco Menzella, Mirco Lusuardi, Carla Galeone, Gloria Montanari, Alberto Cavazza, Nicola Facciolongo

**Affiliations:** 1 Department of Medical Specialties, Pneumology Unit, Arcispedale Santa Maria Nuova-IRCCS, Azienda USL di Reggio Emilia, Via Amendola 2, Viale Risorgimento 56, 42122 Reggio Emilia, Italy; 2 Unit of Respiratory Rehabilitation, Azienda USL di Reggio Emilia, S. Sebastiano Hospital, Correggio, Italy; 3 Department of Pathology, Arcispedale Santa Maria Nuova-IRCCS, Azienda USL di Reggio Emilia, Vial Amendola 2, 42122 Reggio Emilia, Italy

**Keywords:** Bronchial thermoplasty, Severe asthma, Haemoptysis, Necrosis

## Abstract

**Background:**

Bronchial thermoplasty (BT) is an endoscopic procedure for the treatment of severe refractory asthma, based on the local airways delivery of radio-frequency at 65 °C. Several controlled clinical studies demonstrated the effectiveness of BT on clinical outcomes, particularly the reduction of asthma exacerbations. During procedure or shortly after, significant but transient respiratory adverse events have been reported.

**Case report:**

We describe the case of a male, caucasian, 56-year-old, non-smoker patient with non-allergic severe asthma. A few days after the second BT session performed in the left lower lobe, persistent haemoptysis appeared requiring patient hospitalization. A chest CT scan showed mild varicoid bronchiectasis and distal parenchymal infiltrate in the basal anterior segment of the left lower lobe. At fibreoptic bronchoscopy two small nodular neoformations were observed in sub-segmental areas of the same lobe. Histological examination showed mild non-specific inflammation of bronchial mucosa, and some large fragments of peribronchial pulmonary parenchyma with an area of haemorrhagic necrosis. The patient was treated empirically with co-amoxiclav, azithromycin and prednisone. A new chest CT showed a complete resolution of the parenchymal opacity. Finally, the patient underwent the third session of BT, without recurrence of haemoptysis or radiological changes.

**Discussion:**

Bronchial thermoplasty is a generally safe procedure. To our knowledge this is the first report of necrosis of the treated bronchus and haemoptysis complicating BT after the second session. The pulmonary damage was most likely determined by a thermal shock induced by BT. One hypothesis could be a structural fragility of the treated bronchus, possibly related to bronchiectasis. A technical malfunction of the BT controller or the catheter, causing an excessive energy delivery could not be excluded. Adverse events following BT deserve particular attention but should not discourage clinicians from the application of this promising procedure.

## Background

Bronchial thermoplasty (BT) is a procedure for the treatment of severe refractory asthma consisting in the endobronchial controlled delivery of thermal energy. The aim of BT is to modify the structure of the airway wall mainly reducing the amount of smooth muscle. The technique is performed with a device called the Alair Bronchial Thermoplasty System (Boston Scientific, Natick, MA, USA), inserting a disposable catheter (2.0 mm diameter) in the operating channel of a fibreoptic bronchoscope [1]. The distal tip contains an expandable four-electrode basket, through which 65 °C radio-frequencies (RF) are delivered in order to treat all visible subsegmental bronchial areas serially. Lower right lobe, lower left lobe, and upper lobes are treated in sequence on different sessions performed at 20-day intervals. The middle lobe is not treated due to the possible risk of bronchial stenosis because of its small diameter [2]. To date, the use of BT has not been accepted yet on a large scale, due to some controversies on its efficacy and safety profile. According to current knowledge, the basic assumption is that BT can denature and destroy airways smooth muscle (ASM) allowing the reduction of bronchospasm, with clinical improvement in the control of severe asthma symptoms. Several controlled clinical studies (AIR, AIR2, RISA) demonstrated the effectiveness of BT on clinical outcomes, particularly the reduction of asthma exacerbations [1]. Until now, the safety profile of BT has not generated any particular concern, however, data on the long-term safety are still limited. In particular, early doubts about consequences of thermal damage, such as bronchial stenosis, and bronchomalacia, remain to be clarified. During procedure or shortly after, significant but transient respiratory adverse events have been described, e.g. bronchospasm, upper and lower respiratory tract infection, recurrent relapsing atelectasis, and haemoptysis [3].

## Case report

On March 2017, a male, caucasian, 56-year-old, non-smoker patient came to our observation. He was employed as a truck driver. Remote clinical history included hepatitis, nasal polyposis treated with functional endoscopic sinus surgery (FESS), chronic sinusitis, gastroesophageal reflux disease (GERD) undergone laparoscopic GERD surgery (fundoplication). In 2016, a diagnosis of non-allergic asthma was made. Skin prick tests were negative. Respiratory function tests showed a moderate obstruction (FEV_1_ 2.20 L, 68% of predicted; FEV_1_/FVC 67%) without bronchial reversibility after 400 μg of inhaled salbutamol. A few months later, after normalization of lung function parameters following maximal therapy, a positive bronchial provocation test with methacholine showed a degree of bronchial hyperresponsiveness congruent with the diagnosis of bronchial asthma (PDV20 FEV_1_ 136 mcg). Antineutrophil cytoplasmic antibodies (ANCA) were negative. The therapy included formoterol/fluticasone metered-dose inhaler 250/10 μg, two inhalations twice daily and as needed (twice a day on an average), tiotropium bromide 2.5 μg soft mist inhaler, montelukast 10 mg/day. Due to frequent exacerbations and poor control of asthma, systemic corticosteroids (either oral or parenteral) had to be prescribed for over 6 months and 7 unscheduled visits were required in the previous year.

**Fig. 1:**
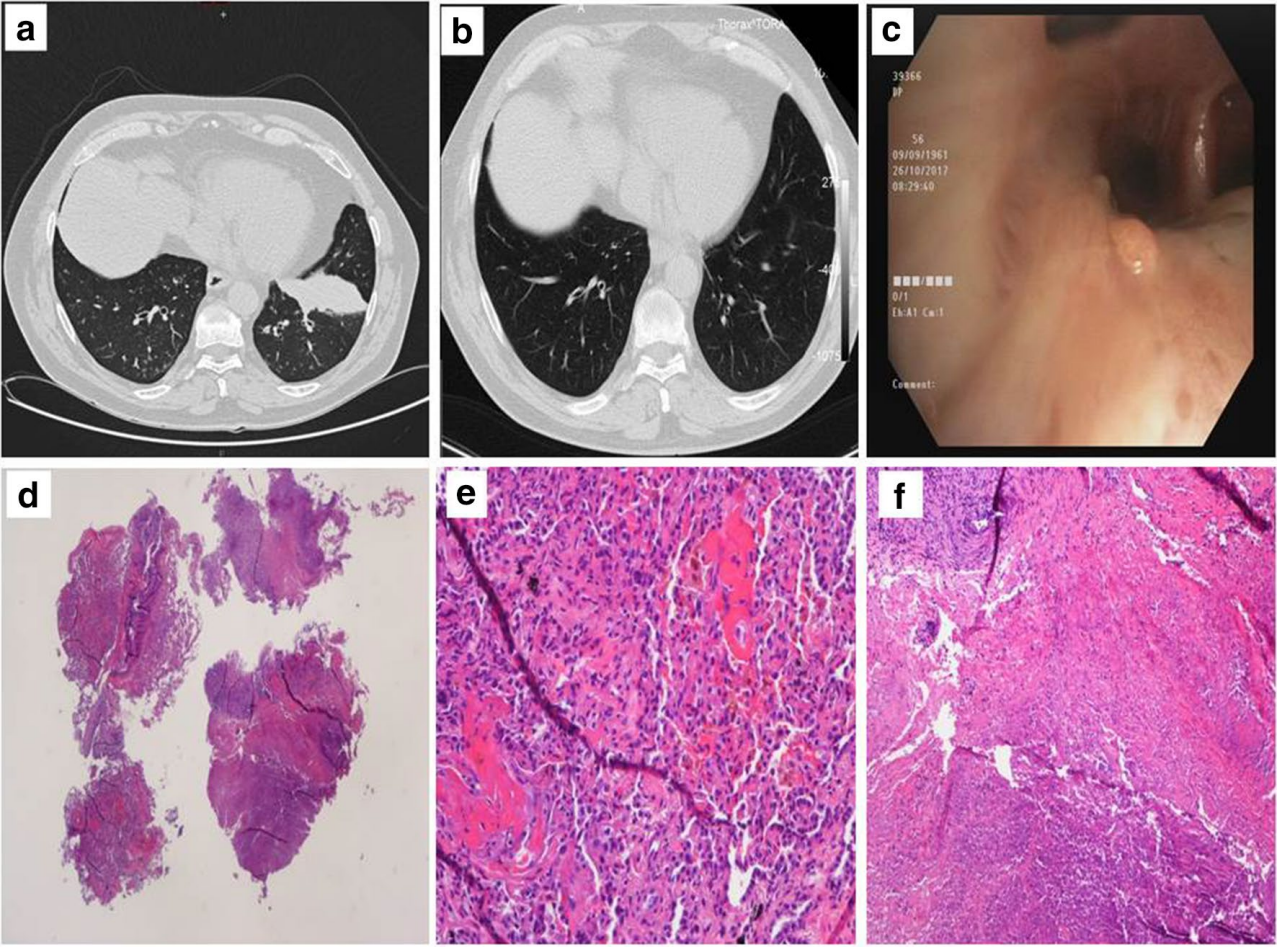
**a** CT image showing the presence of varicoid bronchiectasis and parenchymal infiltration around the basal anterior segmental branch line of the left lower lobe. **b** CT image showing resolution of parenchymal opacity. **c** Fibreoptic bronchoscopy showing two small nodular neoformations in the sub-segmental branches of the lower right lobe. **d** Biopsies (×20 magnification): on the right there are two fragments with necrosis, on the left two fragments with haemorrhage, in the form of blood with fibrin, hemosiderin and organizing pneumonia. **e** and **f** Necrosis and bleeding are best seen at higher magnification, ×200 left and ×100 right, respectively

On February 2017 a chest high-resolution computed tomography (CT) scan showed only mild fibrotic scarring in the anterior basal segment of the lower right lung lobe. On September 2017 the patient received a first session of BT, in the lower right lobe, without any tolerability problem. Three days before the procedure systemic oral corticosteroids (prednisone 50 mg/day) were administered according to usual protocol, to control potential exacerbation of airway inflammation. Immediately before bronchoscopy, inhaled bronchodilators (nebulized salbutamol and ipratropium bromide) were given, along with nebulized lidocaine to provide topical anesthesia. Atropine 0.4–0.6 mg was administered intravenously 15–30 min before the procedure to minimize secretions. According to our procedure, the patient is placed under moderate sedation, a peripheral intravenous (IV) line is taken and supplemental oxygen is administered. The bronchoscope is positioned at the first treatment site, usually the most distal airway in the targeted lobe and the Alair Catheter is introduced through the working channel. The electrode array at the tip of the Catheter is expanded to contact the airway wall. The bronchoscopist activates the RF Controller to deliver low-power, temperature-controlled RF energy to the airway; the energy transfer ends automatically upon completion of the cycle (about 10 s). A single activation of the Catheter delivers RF energy over a distance of approximately 5 mm. After each procedure there is a 1–2 days hospitalization with monitoring of vital parameters and control of chest radiography. On October 5, 2017 a second BT session was performed in the left lower lobe, according to the same procedure. On October 23, persistent haemoptysis appeared requiring patient admission to our ward. On physical examination there was a reduction of breath sounds at the left pulmonary base. The patient underwent a new chest CT scan: around the basal anterior segmental branch line of the left lower lobe, mild varicoid bronchiectasis and distal parenchymal infiltrate were present, in addition to mild signs of airway inflammation in the right lower lobe (Fig. 1a). A fibreoptic bronchoscopy showed two small nodular neoformations in the sub-segmental branches of the same lobe (Fig. 1b). Endobronchial ultrasound (EBUS) with radial probe exploration of the left lower lobar bronchus was also performed with evidence of peripheral infiltrate, sampled with four trans-bronchial biopsies. These changes were not present on endoscopy at the time of the BT procedure, and were detected only after the appearance of haemoptysis.

On histological examination bronchial mucosa showed mild non-specific inflammation. Some large fragments of peribronchial pulmonary parenchyma (Fig. 1d) presented an area of haemorrhagic necrosis, with blood and fibrin, hemosiderin deposits and organizing pneumonia (Fig. 1d, e). No neoplastic cells were observed. Infectious agents were excluded after histochemical staining with Grocott and Ziehl–Nielsen methods. Microbiological cultures for aerobe and anaerobe bacteria were negative as well as cultures for mycobacteria. The patient was treated empirically with co-amoxiclav 1 g three times and azithromycin 500 mg daily for 6 days and prednisone 25 mg per day for 10 days. On December 18, a new chest CT showed a complete resolution of the parenchymal opacities (Fig. 1f). On January 9, 2018, the patient underwent the third session of BT, without recurrence of haemoptysis or subsequent radiological changes. Before performing the last BT session a fibreoptic bronchoscopy showed a complete resolution of the previously reported endoscopic findings.

## Discussion

Bronchial thermoplasty is generally a safe treatment in the long term; no cases of bronchial stenosis in particular have ever been described following BT [2]. On the contrary, during the procedure or shortly after, a significant increase has been reported in respiratory adverse events such as bronchospasm, upper and lower respiratory tract infection, recurrent atelectasis [4], and haemoptysis [5]. Imaging after BT can be associated with ground-glass opacities that undergo spontaneous resolution without any treatment [6]. Severe or even fatal clinical complications following BT have never been recorded in the international literature.

This is the first report of a patient receiving BT with subsequent necrosis of the treated bronchus and haemoptysis. Despite the emerging use of BT in the treatment of severe asthma, the ASM and bronchial wall response to extreme temperatures remains unclear. In a study on a bovine model [7], the authors evaluated the effects, isometric contraction in particular, of ASM exposure to supraphysiological temperatures, with thermal loads of 37, 55, 65 and 95 °C for 30 s. Tissues treated at 55 or 65 °C did not show morphological changes indicative of necrosis or apoptosis, compared with control tissues at 37 °C. In contrast, most cells exposed for 30 s at 95 °C and evaluated within 30 min exhibited varying degrees of alteration, with necrosis and apoptosis. The heat seems to have the ability to directly interrupt the actin-myosin interactions, probably through a denaturation of the motor protein, with immediate loss of the cellular function within the ASM. In literature there is only one report of a similar event but very mild and without haemoptysis. Actually, the first feasibility study of BT in the human airways [8] showed in six subjects scheduled for lung surgery, histological findings with focal necrosis, thrombosis of perichondral vessels, mild focal necrotic cartilage or peribronchial pneumonitis. Patchy accumulation of inflammatory cells, mostly lymphocytes, was found in interstitial spaces, probably due to heat-induced coagulative necrosis of the parenchymal tissue. These alterations were noted in 16 of the 64 Sections (25%) examined, from subjects without symptoms or haemoptysis. In early animal studies on the application of radio-frequency energy at 55, 65 and 75 °C, in one of two dogs killed 1 week after BT, thickening of the bronchial wall was present at CT examination. Histological analysis of the airways suggested that this was due to edema of the treated area [9]. No parenchymal edema was reported in dogs killed at 6, 12 and 157 weeks after BT.

In a recent work by Debray et al. [6] chest CT was performed the day after each BT session in 13 patients with severe asthma, with an evaluation at 1 month of 15 BT-treated lobes overall from 11 patients. Early peribronchial consolidations and ground-glass opacities were found in all treated lobes on day 1, with lower lobes showing complete collapse in three cases. Mild involvement of an adjacent untreated lobe was observed in 12 out of 38 (32%) treated lobes. Patients were completely asymptomatic, the radiological alterations were unrelated to clinical symptoms and spontaneously disappeared after 1 month. The authors hypothesized that these findings probably reflect alveolar inflammation and oedema due to heat-shock and that the extensive effects of BT on the parenchyma induce structural changes of the small airways, resulting in improved asthma control. This hypothesis was however not confirmed by histological data. Another study reported four different radiological patterns of acute radiological abnormalities after BT: peribronchial consolidations with surrounding ground glass opacities (94%), atelectasis (38%), partial bronchial occlusions (63%), and bronchial dilatations (19%) [10].

Thermal coagulation necrosis, or “ablation zone”, typically manifests as a round or oval defect area at CT. Within the necrotizing area, tissue architecture and cellular components appear quite preserved but enzymatic activity on histochemical analysis is not detectable. Initially, transient periablative hyperemia is present as a reaction to damage, which is gradually replaced by granulation tissue and possible scars as part of the healing process [11].

In our patient inflammatory necrotizing and hemorrhagic lung disease appeared 18 days after the second BT session and the only clinical manifestation was haemoptysis. After the first and third sessions there were no problems. The pulmonary damage was most likely determined by a thermal shock induced by BT, with complete resolution after therapy, as confirmed by the chest CT. Identifying specific causes is not at all simple. One hypothesis could be a structural fragility of the treated bronchus or a technical malfunction of the BT controller or the catheter, causing an excessive energy supply. Also the appearance of bronchiectasis, not identified in the pre-procedure CT, can be a consequence of BT as already reported in literature [10].

Necrotizing pneumonia with or without lung abscess is frequently caused by bacteria, in particular anaerobic or facultatively anaerobic ones like *S. aureus*, *K. Pneumoniae* and *S. Pyogenes* [12]. In our case microbiological tests were negative.

Although heat-induced focal necrosis of lung tissue was noted after BT in previous studies, to our knowledge this is the first report of clinically significant necrosis of the pulmonary parenchyma with haemorrhage and haemoptysis. Adverse events following BT deserve particular attention even in case of low risk, but should not discourage clinicians from its application for the increasing evidence of BT efficacy in the long-term control of severe asthma.

## Abbreviations

BT: bronchial thermoplasty
ASM: airway smooth muscle
FESS: functional endoscopic sinus surgery
GERD: gastroesophageal reflux disease
CT: computed tomography
EBUS: endobronchial ultrasound
